# The association between edentulism and chronic kidney disease with mortality: results from the NHANES study (2009–2020)

**DOI:** 10.1186/s12903-025-07166-w

**Published:** 2025-12-01

**Authors:** Huaxiang Jiang, Liping Yin, Zihao Chen, Le Gan, Zishun Qin

**Affiliations:** 1 School/Hospital of Stomatology, Lanzhou University, Lanzhou, 730000 China; 2 The First Clinical Medical College, Lanzhou University, Lanzhou, 730000 China; 3 Jinxian County Traditional Chinese Medicine Hospital, Fuzhou City, Jiangxi Province 344800 P.R. China; 4 Department of Stomatology, The Second Affiliated Hospital, Jiangxi Medical College, Nanchang University, Jiangxi, 330000 China

**Keywords:** NHANES, Edentulism, CKD, Relevance, Mortality

## Abstract

**Background:**

Few studies have examined the association of edentulism and chronic kidney disease (CKD). This study aimed to investigate the association of edentulism and CKD, as well as the association of edentulism and all-cause mortality.

**Methods:**

This study analysed 19,427 patients with varying degrees of edentulism from the National Health and Nutrition Examination Survey (NHANES) from 2009 to 2020. The endpoints were all-cause mortality and CKD, which were determined through the National Death Index (NDI). CKD was calculated based on the estimated glomerular filtration rate (eGFR) and urinary albumin-to-creatinine ratio. A logistic regression classification model and interaction test were used to determine the association of edentulism and CKD. Kaplan–Meier survival analysis, multivariate Cox regression survival models, and stratified analyses were used to explore the correlation between edentulism and mortality risk.

**Results:**

During the follow-up period that included the data of 19,427 individuals, a total of 1,579 cases of all-cause mortality were recorded, representing an incidence of 8.13%. Among these, 865 cases, accounting for 54.78% of the total mortality, were attributed to chronic kidney disease (CKD). After multivariable-adjusted logistic regression analysis, the risk of CKD increased by 39% among participants with complete edentulism (OR 1.39, 95% CI 1.17 ~ 1.66; *P* < 0.001).

Following multivariable-adjusted Cox regression models, a significant connection was identified between edentulism and mortality due to CKD or all causes. Compared with participants without tooth loss, those with maxillary tooth loss had a significantly increased all-cause mortality rate of 61% (HR 1.61, 95% CI 1.30 ~ 1.98, *p* < 0.001), and the CKD-related mortality rate was significantly elevated by 45% (HR 1.45, 95% CI 1.11 ~ 1.90, *p* = 0.007). Participants with both maxillary and mandibular tooth loss presented a significant increase in the all-cause mortality rate of 102% (HR 2.02, 95% CI 1.73 ~ 2.35, *p* < 0.001). The CKD mortality rate was significantly increased by 69% (HR 1.69, 95% CI 1.33 ~ 2.14, *p* < 0.001).

**Conclusion:**

Complete loss of both maxillary and mandibular dentition not only increases the prevalence of CKD but also increases the all-cause mortality and the mortality rates associated with CKD.

**Supplementary Information:**

The online version contains supplementary material available at 10.1186/s12903-025-07166-w.

## Clinical significance

This study provides clinicians with evidence that an increased degree of tooth loss is significantly related to the risk of mortality. Therefore, when assessing the survival status of patients with CKD, attention should be given to tooth loss and oral health.

## Introduction

CKD is a global public health issue, with an increasing incidence worldwide, that affects millions of individuals and causes serious harm to the health and the quality of life of affected individuals [1–3]. In addition, CKD presents significant challenges to health care systems and patient prognosis. CKD is characterized by gradual abnormalities in kidney function and structure over the course of 3 months, leading to a range of complications and increased mortality [4, 5]. Early identification and intervention are crucial for managing CKD and improving patient outcomes [6, 7].

Tooth loss, particularly complete edentulism, is a common oral health problem with an impact that may extend beyond the oral cavity itself. Previous research has indicated a certain relationship between oral health status and several chronic diseases, including periodontal disease, cardiovascular diseases, and diabetes [8, 9]. Edentulism, as a more severe oral health issue, may affect systemic health through various mechanisms [10]. On one hand, edentulism may lead to a decline in masticatory function, subsequently affecting nutritional intake and digestion, adversely impacting the body's metabolic regulation and immune system function [11, 12]. On the other hand, edentulism may be related to a chronic inflammatory state, which is a common pathophysiological basis for various chronic diseases, including CKD [13, 14]. Although previous studies have suggested a link between oral health and general diseases, few studies have specifically investigated the association of edentulism and CKD [15], raising concerns regarding this knowledge gap. Understanding this relationship is essential for developing comprehensive public health strategies that encompass both oral and systemic health. Furthermore, this understanding may provide insight into the mechanisms of the association of oral health and kidney health, creating new avenues for prevention and treatment.

Therefore, this study aimed to investigate the association of edentulism and CKD and the association of edentulism and all-cause mortality through statistical analysis of data from the NHANES.

## Methods

### Data collection from the NHANES

The NHANES is a nationally representative survey conducted by the National Center for Health Statistics (NCHS) of the USA Centers for Disease Control and Prevention (CDC). Its main purpose is to examine the health and dietary conditions of noninstitutionalized individuals throughout the United States. Data were obtained via a variety of methods, including structured household interviews, visits to mobile inspection centres, and laboratory tests, using a complicated, stratified, multistage probabilistic sampling design.The protocol of NHANES was approved by the National Centre for Health Statistics (NCHS) ethics review board, and all participants had provided written informed consent.

**Fig. 1:**
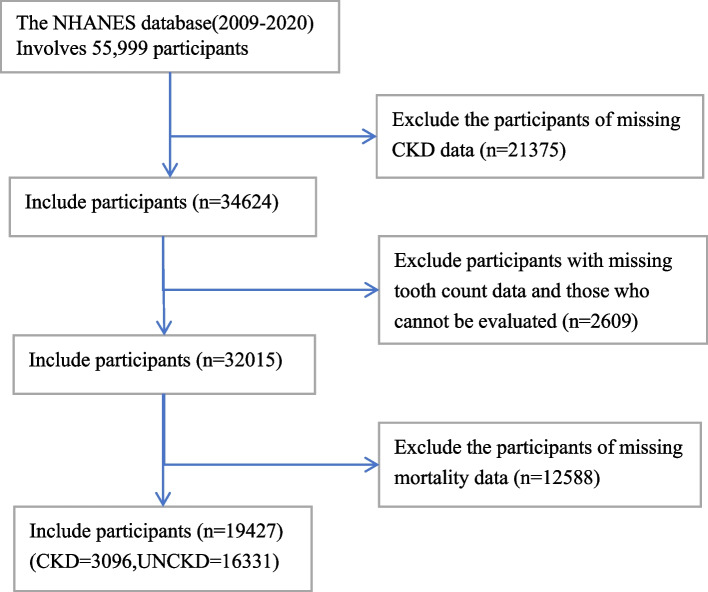
Flowchart of the participants being analysed

The data for this cross-sectional study were obtained from the NHANES database and cover five cycles of the survey (2009–2010, 2011–2012, 2013–2014, 2015–2016, and 2017–2020). Mortality outcomes were determined using records from the NDI, with follow-up extending to the last available update. A total of 55,999 participants aged 18 years or older were initially included. Individuals were excluded if they met the following criteria: (1) had missing chronic kidney disease (CKD) data (*n* = 21,375); (2) lacked tooth count data and were unable to be assessed (*n* = 2,609); or (3) had missing mortality data (*n* = 12,588). Ultimately, this study included 19,427 participants (see Fig. 1).

### Exposure variable: edentulism

During clinical examinations, trained and calibrated dentists assessed the number of dental arches and noted the number of all teeth in the participants' mouths. The gold standard examination was conducted to observe the onsite operations, with dental checks repeated 20 to 25 times during each visit. If the correlation between raters was not within an acceptable range, onsite retraining would be conducted, and future monitoring by onsite reviewers would be reinforced. Approximately 10% of the examined participants were asked to return for repeat checks. The purpose of these repeat checks was to monitor the internal consistency of the reviewers regarding the data collection process. Each dental examiner participated in a retraining course once a year, which was also conducted by the gold standard examiner.

This study strictly adheres to the NHANES protocol, defining edentulism as a state of complete absence of natural teeth in a dental arch, regardless of whether it has been restored with implants. Therefore, implant-supported dental arches (with no residual natural teeth) are included within the edentulism category. In this study, participants were specifically classified into four types of dental conditions: 1) dentulous individuals: at least one natural tooth present in both the upper and lower jaws; 2) maxillary dentition missing: all natural teeth missing in the upper jaw; 3) mandibular dentition missing: all natural teeth missing in the lower jaw; and 4) complete dentition missing: all natural teeth missing in both the upper and lower jaws.

### Definition of CKD

The eGFR was computed using the Chronic Kidney Disease Epidemiology Collaboration (CKD-EPI) formula [16]: eGFR = 141 × min (Scr/κ, 1)α × max (Scr/κ, 1)−1.209 × 0.993Age × 1.018 [for females], with Scr representing serum creatinine, κ being 0.7 for females and 0.9 for males, α set at −0.329 for females and −0.411 for males, min signifying a lower Scr/κ or 1, and max denoting a greater Scr/κ or 1. In line with previously established guidelines, CKD was defined as the absence of CKD; presence of CKD, indicated by an eGFR < 60 mL/min/1.73 m2 or a UACR > 30 mg/g [17].

### Mortality data

The death data were obtained from the NDI database, which is managed by the Centers for Disease Control and Prevention [https://www.cdc.gov/nchs/data-linkage/mortality-public.htm]. The duration of follow-up was from the date of the initial interview to the time of death or until December 31, 2019, which was the last time the NDI database was updated. Deaths due to CKD were identified using the International Classification of Diseases, Tenth Revision (ICD-10) codes, specifically N02-N03, N05-N07, and N26.

### Covariates

The factors considered in the study included age, sex, race (Hispanic American, non-Hispanic white, non-Hispanic black, and other races), cultural differences (no college, college degree or higher), marital conditions (living with a partner, separated from a partner), a history of diabetes, and a history of hypertension. The criteria of these covariates were detailed on the NHANES website of the Centers for Disease Control and Prevention (CDC) [https://www.cdc.gov/nchs/nhanes/].

### Statistical analysis

Baseline characteristics were delineated in relation to dental status. Continuous variables are represented as the mean ± standard deviation. These variables were compared between the groups using analysis of variance. Categorical variables are presented as proportions. The p value of each categorical variable was obtained using the chi-square test or Fisher’s exact test. Three sets of logistic regression models (Model 1, Model 2, and Model 3) were constructed to explore the associations of edentulism and CKD. Moreover, three sets of multivariate Cox regression models (also named Model 1, Model 2, and Model 3) were established to measure the degree of association of edentulism and all-cause mortality and CKD-related mortality. Model 1 did not adjust for any covariates; Model 2 adjusted for sex and age; and Model 3 adjusted for sex, age, race, and marital condition. Kaplan–Meier survival curves were used to graphically demonstrate the nexus between edentulism, CKD, and mortality by all causes. Additionally, subgroup analyses were conducted based on fully adjusted Cox models to explore the nexus between edentulism and mortality across disparate subgroups.A directed acyclic graph (DAG) (Appendix Fig. 1) provides support for causal hypotheses in statistical analysis, demonstrating the hypothesized causal relationship structure.

All of the means of analysis were performed using Excel and R software (version 4.3.0). *P* < 0.05 was considered indicative of statistical significance.

## Results

### Baseline characteristics of the participants

According to the eligibility criteria of this study, a total of 19,427 individuals were included, with an average age of 47.3 ± 0.32 years. Among the participants, 9,564 were males, accounting for 48.78%, and 9,863 were females, accounting for 51.22%. Table 1 provides a detailed description of the participants. Among these participants, 3,452 had chronic kidney disease, accounting for 14.73%. There were 17,221 dentulous individuals, accounting for 88.64%; 1,248 individuals had complete edentulousness in both jaws, accounting for 6.42%; 890 individuals had missing teeth only in the upper jaw, accounting for 4.58%; and 68 individuals had missing teeth only in the lower jaw, accounting for 0.35%. There were significant intra-group differences in age and race (*P* < 0.05), though the sex did not vary between the groups (P > 0.05).

**Table 1:** Baseline characteristics of participants according to the four-layer classification of edentulism

Variable	Total (*n* = 19,427)	Dentulous individuals (*n* = 17,221)	Maxillary dentition missing (*n* = 890)	Mandibular dentition missing (*n* = 68)	Complete dentition missing (*n* = 1248)	*P*
Age, Mean (SD)	47.35 (± 0.32)	45.65 (± 0.30)	63.62 (± 0.56)	66.97 (± 1.92)	66.51 (± 0.60)	<.001
Average follow-up Time	78.80 (± 1.07)	79.33 (± 1.08)	74.38 (± 2.40)	72.34 (± 4.22)	72.46 (± 1.77)	<.001
Sex, n(%)						0.053
Male	9564 (48.78)	8490 (49.05)	411 (43.96)	39 (55.90)	624 (46.77)	
Female	9863 (51.22)	8731 (50.95)	479 (56.04)	29 (44.10)	624 (53.23)	
Race, n(%)						<.001
Mexican American	2908 (8.56)	2751 (9.09)	74 (3.25)	6 (5.05)	77 (2.68)	
Other Race	4601 (13.69)	4194 (13.91)	164 (10.73)	16 (14.66)	227 (11.71)	
Non-Hispanic White	7811 (66.84)	6723 (66.31)	412 (72.14)	30 (63.36)	646 (73.15)	
Non-Hispanic Black	4107 (10.91)	3553 (10.69)	240 (13.88)	16 (16.93)	298 (12.46)	
Education, n(%)						<.001
Below college	9438 (38.84)	7894 (36.30)	582 (61.02)	53 (80.05)	909 (68.70)	
College Graduate or above	9989 (61.16)	9327 (63.70)	308 (38.98)	15 (19.95)	339 (31.30)	
Marital status,n(%)						<.001
Live together	12,108 (65.68)	11,024 (66.93)	478 (57.54)	40 (53.26)	566 (48.41)	
Separated	7319 (34.32)	6197 (33.07)	412 (42.46)	28 (46.74)	682 (51.59)	
Hypertension, n(%)						<.001
No	11,526 (63.03)	10,858 (65.80)	282 (34.11)	25 (35.86)	361 (33.17)	
Yes	7882 (36.97)	6348 (34.20)	605 (65.89)	43 (64.14)	886 (66.83)	
Diabetes, n(%)						<.001
No	15,570 (85.50)	14,257 (87.21)	548 (70.76)	40 (62.73)	725 (64.76)	
Yes	3564 (14.50)	2726 (12.79)	318 (29.24)	27 (37.27)	493 (35.24)	
CKD, n(%)						<.001
No	15,975 (85.27)	14,649 (87.18)	570 (68.97)	42 (59.01)	714 (62.37)	
Yes	3452 (14.73)	2572 (12.82)	320 (31.03)	26 (40.99)	534 (37.63)	

### Association of edentulism and CKD

As shown in Table 2, there was a significant positive correlation between edentulism and CKD (*P* < 0.001). According to the univariate analysis, the risk of CKD in patients whose maxillary dentition was missing was greater than that in dentulous individuals (OR = 3.06, 95% CI:2.49 ~ 3.75, *P* < 0.001), and the risk in patients whose mandibular dentition was missing was greater than that in dentulous individuals (OR = 4.72, 95% CI:2.34 ~ 9.55, *P* < 0.001). Additionally, the risk of CKD in patients with complete dentition missing was greater than that in dentulous individuals (OR = 4.10, 95% CI: 3.40 ~ 4.95; *P* < 0.001). Therefore, a multivariate logistic regression model was used to further investigate the correlation between CKD and edentulism. After adjusting for covariates such as age, sex, race, marital status, and education (Model 2), missing maxillary dentition was positively correlated with CKD (OR = 1.35, 95% CI = 1.07 ~ 1.69, *P* = 0.014), and missing mandibular dentition was significantly associated with CKD (OR = 1.60, 95% CI = 1.07 ~ 1.69, *P* < 0.001). After all of the covariates were adjusted (Model 3), missing maxillary dentition was still associated with CKD (OR = 1.21, 95% CI = 0.96 ~ 1.54), missing mandibular dentition was still associated with CKD (OR = 1.60, 95% CI = 0.79 ~ 3.25), and a significant correlation between missing all dentition and CKD (OR = 1.39, 95% CI = 1.17 ~ 1.66, *P* < 0.001) remained. In addition, the risk significantly increased.

**Table 2:** Logistic regression, multi-model strategy

Variables	Model 1		Model 2		Model 3	
	OR (95% CI)	*P*	OR (95% CI)	*P*	OR (95% CI)	*P*
Edentulous						
Dentulous individuals	1.00 (Reference)		1.00 (Reference)		1.00 (Reference)	
Missing maxillary dentition	3.06 (2.49–3.75)	<.001	1.35 (1.07–1.69)	0.014	1.21 (0.96–1.54)	0.114
Missing mandibular dentition	4.72 (2.34–9.55)	<.001	1.82 (0.92–3.59)	0.092	1.60 (0.79–3.25)	0.201
Missing all dentition	4.10 (3.40–4.95)	<.001	1.60 (1.34–1.91)	<.001	1.39 (1.17–1.66)	<.001

Model 1: Crude

Model 2: Adjusted for sex, age, race, marital status, and education

Model 3: Adjusted for sex, age, race, marital status, education, hypertension, and diabetes

*OR* Odds Ratio, *CI* Confidence Interval

### Subgroup analysis of the association of edentulism and CKD

**Fig. 2:**
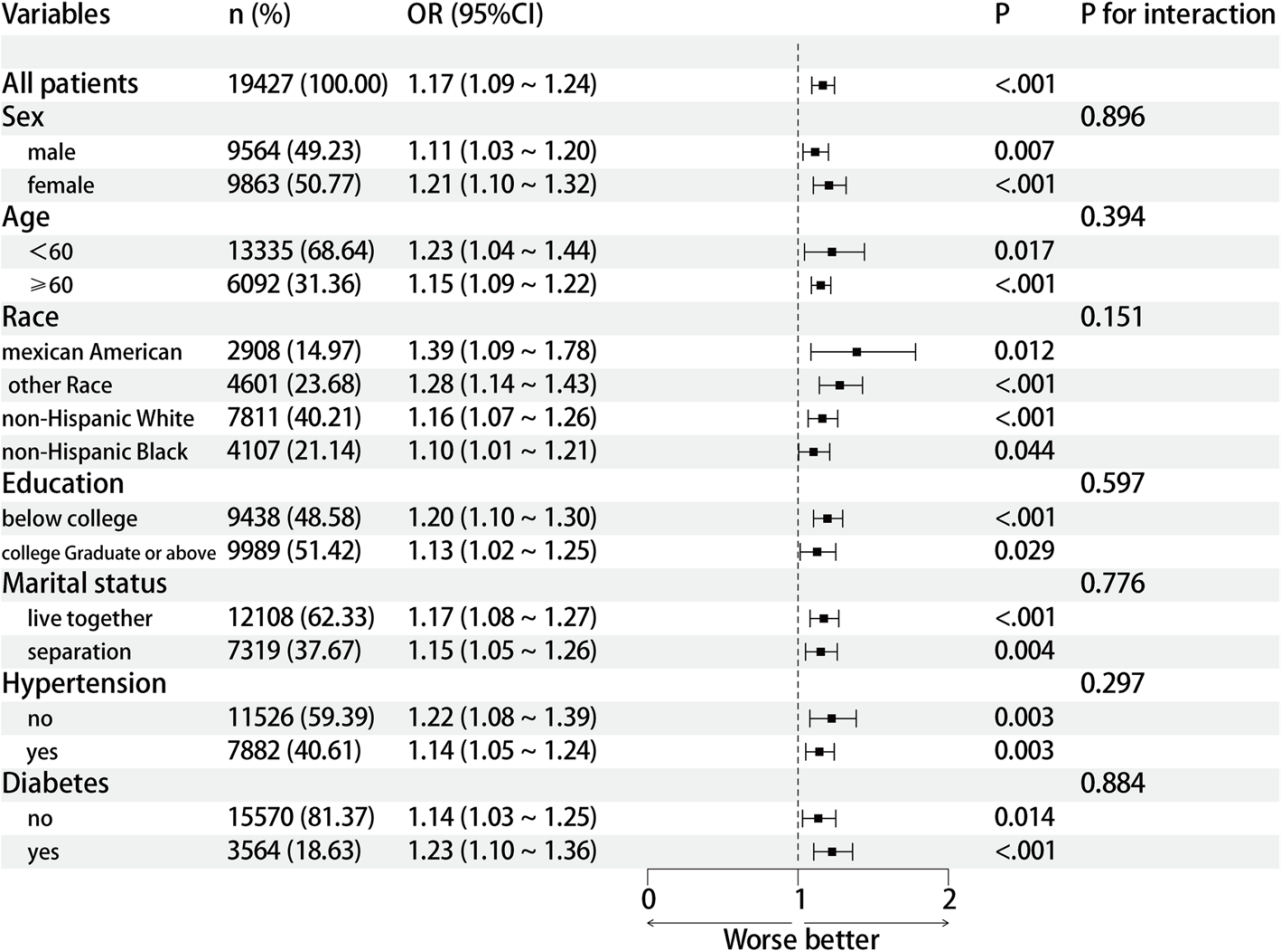
Subgroup analysis of the relationship between edentulism and CKD

To determine whether the association of edentulism and CKD remains throughout the broader population, we conducted subgroup examinations and interaction assessments that focused on age, sex, racial background, cultural aspects, marital conditions, blood pressure conditions, and diabetic status. Our results suggest that this connection is not constant across all groups. A detailed subgroup analysis exploring the relationship of edentulism and CKD is presented in Fig. 2. When a range of influencing factors was accounted for, a notable association of edentulism and CKD emerged (*P* < 0.05). The positive relationship between edentulism and CKD is not affected by variables such as age, sex, race, cultural nuances, marital situation, high blood pressure, or diabetes. Moreover, no significant interactions were detected(all *P* > 0.05).

### Relationship between edentulism and mortality rate

During the follow-up period, which included 19,427 individuals, 1,579 cases (8.13%) of all-cause mortality were reported, including 865 (54.78%) due to CKD. The results of the univariate and multivariate Cox proportional hazards models are shown in Appendix 3.3, indicating that age, sex, marital status, hypertension, diabetes, edentulism, and CKD are risk factors for mortality (*P* < 0.05). The mortality risk was distinctly increased in older individuals, males, those who were separated, and those with hypertension, diabetes, edentulism, or CKD. The hazard ratio (HR) for mortality associated with edentulism was 1.85 (1.57–2.17), whereas the HR for CKD was 2.23 (1.98–2.51).

**Fig. 3:**
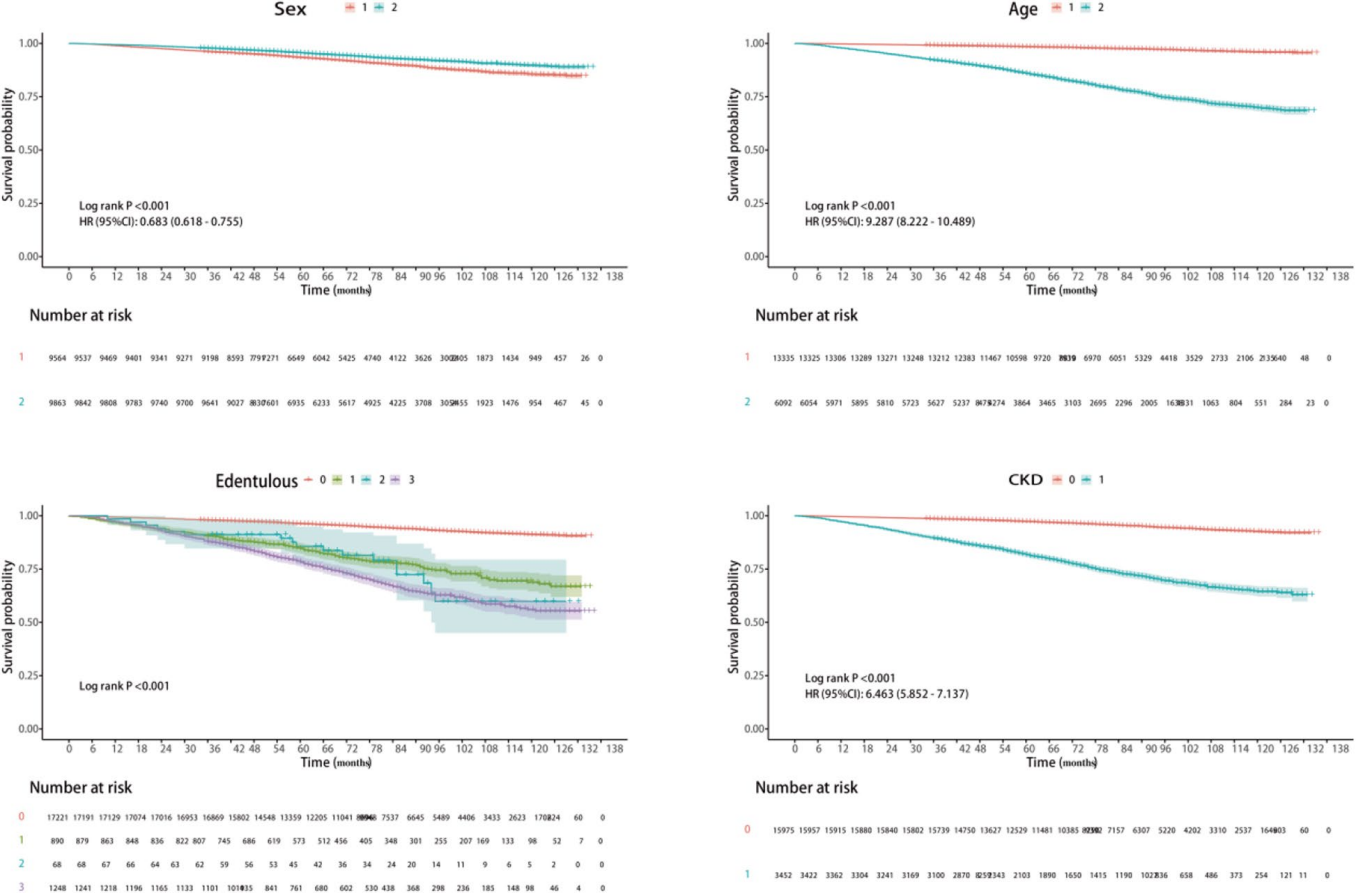
Comparison of survival curves (KM) for sex (1 stands for male, 2 stands for female), age (1 means less than 60 years old, 2 means 60 years or older), edentulism (0 represents dentulous individuals, 1 represents missing maxillary dentition, 2 represents missing mandibular dentition, 3 represents missing all dentition), and CKD (0 represents no, 1 represents yes)

According to the fully adjusted model presented in Table 3, participants with maxillary edentulousness had a significantly increased all-cause mortality rate of 61% (HR = 1.61, 95% CI = 1.30–1.98, *P* < 0.001) compared with those without edentulousness. The CKD-related mortality rate was also significantly increased by 45% (HR = 1.45, 95% CI = 1.11–1.90, *P* = 0.007). Participants with both maxillary and mandibular edentulousness had a significantly increased all-cause mortality rate of 102% (HR = 2.02, 95% CI = 1.73–2.35, *P* < 0.001), and the CKD-related mortality rate also significantly increased by 69% (HR = 1.69, 95% CI = 1.33–2.14, *P* < 0.001). Figure 3 shows the results of the Kaplan–Meier survival analysis, which revealed that older age, male sex, complete edentulism, and the presence of CKD were associated with the highest all-cause mortality rate (log-rank *P* < 0.001).

**Table 3:** HRs (95% CIs) for mortality according to the edentulism index

	**Edentulous**			
	Dentulous individuals	Missing maxillary dentition	Missing mandibular dentition	Missing all dentition
	HR (95% CI) *P*	HR (95% CI) *P*	HR (95% CI) *P*	HR (95% CI) *P*
All-cause mortality				
Model 1	1.00 (Reference)	4.59 (3.75 5.62) <.001	6.49 (3.87–10.88) <.001	7.14 (6.21–8.22) <.001
Model 2	1.00 (Reference)	1.77 (1.44–2.16) <.001	1.91 (1.23–2.97)0.004	2.33 (2.01–2.70) <.001
Model 3	1.00 (Reference)	1.61 (1.30–1.98) <.001	1.70 (1.13–2.56)0.012	2.02 (1.73–2.35) <.001
CKD-related mortality				
Model 1	1.00 (Reference)	2.59 (2.04–3.30) <.001	3.18 (1.41–7.15)0.005	3.25 (2.56–4.12) <.001
Model 2	1.00 (Reference)	1.48 (1.15–1.91)0.002	1.88 (0.93–3.80)0.078	1.73 (1.38–2.18) <.001
Mode l3	1.00(Reference)	1.45 (1.11–1.90)0.007	1.78 (0.88–3.59)0.110	1.69 (1.33–2.14) <.001

Model 1: Crude

Model 2: Adjusted for sex and age

Model3: Adjusted for sex, age, race, education, and marital status

*HR* Hazard Ratio, *CI* Confidence Interval

### Interaction test of edentulism and mortality rate

In Appendix 3.5, the HR for edentulism and all-cause mortality among all patients was 1.34, with a 95% CI of 1.27–1.41 and a *P* < 0.001, indicating a significant connection between edentulism and mortality due to all causes. The risk of mortality from all causes was increased in edentulous patients. In age stratification, the P value for the interaction test was less than 0.001, suggesting a significant interaction effect of age on the association of edentulism and all-cause mortality, with notable risk differences across different age groups. In the racial stratification, the P value for the interaction test was 0.001, indicating a significant interaction effect of race on the association of edentulism and all-cause mortality, with significant risk differences among different racial groups. In stratifications for hypertension and diabetes, the interaction test *P* values were < 0.001 and 0.015, respectively, indicating significant interactions of hypertension and diabetes status on the association of edentulism and all-cause mortality, with a more pronounced risk in the group without hypertension and diabetes. These findings highlight that the effects of different population characteristics on the relationship between edentulism and all-cause mortality varies.

In Table 4, the HR for edentulism and CKD mortality was 1.25 among all patients, with a 95% CI of 1.15–1.35 and a *P* < 0.001, indicating a significant association of edentulism and CKD-related mortality, with a markedly increased risk of CKD mortality in edentulous patients. These results remained consistent across different subgroups, demonstrating a positive correlation between edentulism and both types of mortality.The positive relationship between edentulism and CKD mortality was not affected by variables such as age, sex, race, cultural nuances, marital situation, high blood pressure, or diabetes. Because no significant interactions were detected(all *P* > 0.05).

**Table 4:** Stratified analyses of the associations of edentulism and CKD-related mortality

Variables	n (%)	HR (95% CI)	*P*	P for interaction
All patients	3096 (100.00)	1.25 (1.15–1.35)	<.001	
Sex				0.321
Male	1321 (42.67)	1.27 (1.12–1.43)	<.001	
Female	1775 (57.33)	1.22 (1.11–1.34)	<.001	
Age				0.332
< 60 years	1157 (37.37)	1.43 (0.98–2.07)	0.062	
≥ 60 years	1939 (62.63)	1.23 (1.13–1.33)	<.001	
Race				0.072
Mexican American	400 (12.92)	1.16 (0.96–1.40)	0.126	
Other Race	1285 (41.51)	1.25 (1.13–1.39)	<.001	
Non-Hispanic White	835 (26.97)	1.18 (1.04–1.33)	0.009	
Non-Hispanic Black	576 (18.60)	1.44 (1.15–1.81)	0.001	
Education				0.121
Below college	1746 (56.40)	1.20 (1.08–1.33)	<.001	
College graduate or above	1350 (43.60)	1.34 (1.21–1.49)	<.001	
Marital status				0.056
Live together	1668 (53.88)	1.36 (1.19–1.56)	<.001	
Separated	1428 (46.12)	1.16 (1.05–1.29)	0.004	
Hypertension				0.054
No	908 (29.35)	1.36 (1.15–1.61)	<.001	
Yes	2186 (70.65)	1.22 (1.11–1.35)	<.001	
Diabetes				0.509
No	1794 (59.05)	1.24 (1.11–1.39)	<.001	
Yes	1244 (40.95)	1.24 (1.11–1.38)	<.001	

*HR* Hazard Ratio, *CI* Confidence Interval

## Discussion

This study used a large population sample from the NHANES (2009–2020) to investigate the relationships between edentulism, CKD, and mortality. While previous research has established a qualitative link between periodontal disease/tooth loss and CKD [18], critical gaps remain in understanding the specific impact of edentulism severity on CKD prevalence and, more importantly, on mortality outcomes (both all-cause and CKD-specific). Furthermore, the robustness of this association across diverse populations and the underlying biological pathways of the associations of oral health catastrophes, such as edentulism, to systemic kidney dysfunction and death require further exploration. This study directly addresses these gaps by providing precise quantitative risk estimates for different edentulism types on CKD prevalence and mortality, conducting comprehensive subgroup analyses to assess consistency, and integrating visualization of survival dynamics while also delving deeper into potential mechanistic links.

Previous studies have focused primarily on qualitative associations or periodontal disease in general, with limited quantification of the magnitude of risk posed specifically by complete edentulism and sparse data regarding its impact on mortality endpoints [19]. This study used multivariable-adjusted logistic and Cox regression models to precisely quantify the significantly elevated risks associated with different edentulism types: CKD prevalence increased by 39% (OR 1.39, 95% CI = 1.17 ~ 1.66; *P* < 0.001) in completely edentulous participants. More significantly, we demonstrated, for the first time in a large national cohort, the profound mortality impact: all-cause mortality significantly increased by 102% (HR 2.02, 95% CI = 1.73–2.35, *P* < 0.001), as was CKD-specific mortality by 69% (HR 1.69, 95% CI = 1.33–2.14, *P* < 0.001) in participants who were edentulous in both jaws. This quantification of mortality risk, particularly CKD-related mortality, represents a substantial advancement beyond previous work focused primarily on prevalence or surrogate markers, providing clinicians with concrete data for risk stratification.

### Synergy in clinical diagnosis and treatment models

In clinical practice, this study encourages physicians to shift their diagnostic and treatment thinking, incorporating oral health as an important component of the patient physical health assessments. During patient consultations, physicians should not only focus on oral symptoms but also be vigilant about the potential kidney disease risks that may be associated with edentulism, achieving collaborative diagnosis and treatment among multiple disciplines, such as dentistry and nephrology [20, 21]. For example, when providing oral restoration or treatment for edentulous patients, simultaneous kidney function monitoring can be conducted to identify and manage potential kidney issues in a timely manner [22]. During the management of patients with kidney disease, attention should be given to the oral health status, and targeted oral care measures should be implemented to improve patients' quality of life and prognosis.

### Diversification of scientific research directions

This study offers new ideas and directions for further exploration of the potential mechanisms of the associations of edentulism, CKD and mortality. Factors such as chewing dysfunction, inadequate nutritional intake, and oral microbiome dysbiosis caused by edentulism may affect kidney health through various pathways, such as triggering chronic inflammatory responses, promoting oxidative stress, and interfering with endocrine metabolism [23]. Future research can explore these potential mechanisms from multiple perspectives, including molecular biology, cell biology, and immunology, laying the foundation for the development of new diagnostic markers, therapeutic targets, and intervention methods and promoting the interdisciplinary integration and development of related fields.

Collectively, the compelling evidence of significant mortality risk presented here has the potential to reshape public perception. Edentulism is no longer merely a dental concern, but can now be viewed as a critical indicator of overall health vulnerability. This shift can empower individuals to prioritize oral health maintenance and seek timely dental care while motivating broader societal engagement (such as community programs and policy initiatives) in promoting oral health as an integral component of general well-being and longevity [24, 25].

However, this study also has some limitations. 1)Explanation of sample exclusion: Although this study was based on nationally representative data from the NHANES, to meet specific research objectives (completeness of key variables and clear definition of exposure/outcome), the final sample size was 19,427 participants (accounting for 34.6% of the original sample). Strict exclusion criteria may lead to selection bias and limit the complete generalization of the results to the original sample population. 2)Omission of Potential Confounding Factors: Although common variables, such as age, sex, race/ethnicity, cultural differences, marital condition, diabetes, and hypertension history, were considered, some potential confounding variables, such as patients' dietary habits, smoking and drinking history, cardiovascular diseases and BMI, which may have had some impact on the research results, may have been unaccounted for. Subsequent studies should further refine the selection of covariates and comprehensively collect relevant information to reduce confounding bias and improve the accuracy of research conclusions. As this study includes multiple subgroup analyses and interaction tests, and a large number of hypothesis tests were conducted within these subgroups with relatively low clinical relevance, we did not conduct stringent statistical corrections. Consequently, the findings from these subgroup analyses should be interpreted as exploratory and require further validation through future studies with larger sample sizes. 3)Representativeness and Generalizability of the Data: The sample size of the subgroup with missing mandibular dentition was smaller than that of the other subgroups. A small sample size may lead to unstable estimates and a wide confidence interval, resulting in uncertainty in the results and the need for caution in their interpretation. Although NHANES data are nationally representative, they are primarily based on the U.S. population, which may somewhat limit the extrapolation of the results to other countries or regions. Differences in racial composition, lifestyle, and health care systems across different countries and regions may influence the relationships between edentulism, CKD, and mortality. Multicentre studies conducted in different countries or regions are needed, as is incorporating more diverse populations to validate the generalizability of the findings of this study and provide broader references for public health policy formulation and clinical practice on a global scale.

## Conclusion

Edentulism may be a potential risk factor for the increasing prevalence of chronic kidney disease (CKD), and it may be associated with the rise in all-cause mortality and CKD-specific mortality, suggesting a positive correlation between edentulism and the two mortality rates.

## Supplementary Information

Supplementary Material 1.

Supplementary Material 2.

Supplementary Material 3.

Supplementary Material 4.

## Data Availability

The datasets used and analyzed during the current study are publicly available from the National Health and Nutrition Examination Survey (NHANES) through the Centers for Disease Control and Prevention (CDC) website. The data files and related documentation can be accessed at the NHANES website (https://www.cdc.gov/nchs/nhanes/index.html).
