# Supplementary figures and images for: The association between edentulism and chronic kidney disease with mortality: results from the NHANES study (2009–2020)
Source: BMC Oral Health. 2025 Dec 1;26:44. doi: 10.1186/s12903-025-07166-w (PMC12781603; doi:10.1186/s12903-025-07166-w)

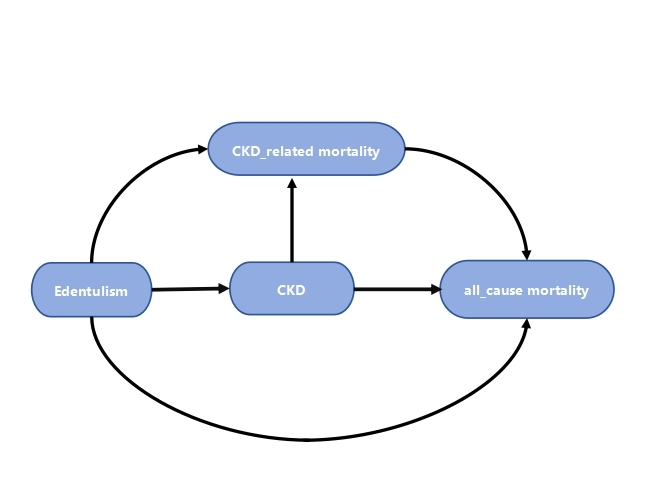

Supplement: Supplementary file 1 — Supplementary Material 1. [file 12903_2025_7166_MOESM1_ESM.jpg]
