# Supplementary material for: The association between edentulism and chronic kidney disease with mortality: results from the NHANES study (2009–2020)
Source: BMC Oral Health. 2025 Dec 1;26:44. doi: 10.1186/s12903-025-07166-w (PMC12781603; doi:10.1186/s12903-025-07166-w)
Supplement: Supplementary file 2 — Supplementary Material 2. [file 12903_2025_7166_MOESM2_ESM.docx]

**Edentulism**

**CKD_related mortality**

**all_cause mortality**

**CKD**

Edentulism may be a potential risk factor for the increasing prevalence of chronic kidney disease (CKD), and it may be associated with the rise in all-cause mortality and CKD-specific mortality, suggesting a positive correlation between edentulism and the two mortality rates.
