# Supplementary material for: The association between edentulism and chronic kidney disease with mortality: results from the NHANES study (2009–2020)
Source: BMC Oral Health. 2025 Dec 1;26:44. doi: 10.1186/s12903-025-07166-w (PMC12781603; doi:10.1186/s12903-025-07166-w)
Supplement: Supplementary file 3 — Supplementary Material 3. [file 12903_2025_7166_MOESM3_ESM.docx]

appendix 3.3, Results of the univariate and multivariate Cox proportional hazards models

| **Variables** | **univariate** | | **multivariate** | | | |
| --- | --- | --- | --- | --- | --- | --- |
|  | ***P*** | **HR (95%CI)** | |  | ***P*** | **HR (95%CI)** |
| **Age** | <.01 | 1.09 (1.09 - 1.10) | |  | <.01 | 1.07 (1.06 - 1.07) |
| **Sex** |  |  | |  |  |  |
| male |  | 1.00 (Reference) | |  |  | 1.00 (Reference) |
| female | <.01 | 0.82 (0.74 - 0.91) | |  | <.01 | 0.61 (0.54 - 0.69) |
| **Race** |  |  | |  |  |  |
| mexican American |  | 1.00 (Reference) | |  |  | 1.00 (Reference) |
| other Race | 0.82 | 1.04 (0.75 - 1.43) | |  | 0.09 | 0.79 (0.60 - 1.04) |
| non-Hispanic White | <.01 | 2.27 (1.71 - 3.00) | |  | 0.15 | 1.20 (0.94 - 1.53) |
| non-Hispanic Black | <.01 | 1.81 (1.34 - 2.44) | |  | 0.92 | 0.99 (0.76 - 1.28) |
| **Marital status** |  |  | |  |  |  |
| live together |  | 1.00 (Reference) | |  |  | 1.00 (Reference) |
| separation | <.01 | 1.92 (1.69 - 2.18) | |  | <.01 | 1.65 (1.42 - 1.91) |
| **Education** |  |  | |  |  |  |
| below college |  | 1.00 (Reference) | |  |  | 1.00 (Reference) |
| college Graduate or above | <.01 | 0.62 (0.53 - 0.72) | |  | 0.07 | 0.88 (0.76 - 1.01) |
| **Hypertension** |  |  | |  |  |  |
| No |  | 1.00 (Reference) | |  |  | 1.00 (Reference) |
| Yes | <.01 | 4.45 (3.87 - 5.11) | |  | <.01 | 1.29 (1.11 - 1.49) |
| **Diabetes** |  |  | |  |  |  |
| No |  | 1.00 (Reference) | |  |  | 1.00 (Reference) |
| Yes | <.01 | 3.42 (3.00 - 3.89) | |  | <.01 | 1.26 (1.08 - 1.46) |
| **Edentulous** |  |  | |  |  |  |
| Dentulous individuals |  | 1.00 (Reference) | |  |  | 1.00 (Reference) |
| maxillary dentition missing | <.01 | 4.59 (3.75 - 5.62) | |  | <.01 | 1.53 (1.23 - 1.90) |
| mandibular dentition missing | <.01 | 6.49 (3.87 - 10.88) | |  | 0.03 | 1.52 (1.05 - 2.20) |
| complete dentition missing | <.01 | 7.14 (6.21 - 8.22) | |  | <.01 | 1.85 (1.57 - 2.17) |
| **CKD** |  |  | |  |  |  |
| No |  | 1.00 (Reference) | |  |  | 1.00 (Reference) |
| Yes | <.01 | 6.77 (6.13 - 7.47) | |  | <.01 | 2.23 (1.98 - 2.51) |

Among the follow-up of 19,427 individuals, there were 1,579 cases (8.13%) of all-cause mortality, of which 865 individuals (54.78%) died from CKD. The results of the univariate and multivariate Cox proportional hazards models were shown in Appendix 3.3, indicating that age, gender, marital status, hypertension, diabetes, edentulism, and CKD are risk factors for mortality (P < 0.05). The mortality risk was distinctly increased in older individuals, males, those who are separated, and those with hypertension, diabetes, edentulism, and CKD. The hazard ratio (HR) for mortality associated with edentulism was as high as 1.85 (1.57 - 2.17), while the HR for CKD was 2.23 (1.98 - 2.51).
