# Supplementary material for: The association between edentulism and chronic kidney disease with mortality: results from the NHANES study (2009–2020)
Source: BMC Oral Health. 2025 Dec 1;26:44. doi: 10.1186/s12903-025-07166-w (PMC12781603; doi:10.1186/s12903-025-07166-w)
Supplement: Supplementary file 4 — Supplementary Material 4. [file 12903_2025_7166_MOESM4_ESM.docx]

appendix 3.5, Stratified analyses of the connections between edentulism and Mortality due to all causes

| Variables | n (%) | HR (95%CI) | *P* | P for interaction |
| --- | --- | --- | --- | --- |
|  |  |  |  |  |
| All patients | 19427 (100.00) | 1.34 (1.27 ~ 1.41) | <.001 |  |
| **sex** |  |  |  | 0.085 |
| male | 9564 (49.23) | 1.27 (1.18 ~ 1.36) | <.001 |  |
| female | 9863 (50.77) | 1.41 (1.31 ~ 1.50) | <.001 |  |
| **age** |  |  |  | <.001 |
| <60 | 13335 (68.64) | 1.67 (1.43 ~ 1.96) | <.001 |  |
| ≥60 | 6092 (31.36) | 1.31 (1.24 ~ 1.38) | <.001 |  |
| **race** |  |  |  | 0.001 |
| mexican American | 2908 (14.97) | 1.22 (1.01 ~ 1.47) | 0.040 |  |
| other Race | 4601 (23.68) | 1.67 (1.45 ~ 1.92) | <.001 |  |
| non-Hispanic White | 7811 (40.21) | 1.34 (1.26 ~ 1.43) | <.001 |  |
| non-Hispanic Black | 4107 (21.14) | 1.19 (1.07 ~ 1.32) | 0.001 |  |
| **Education** |  |  |  | 0.070 |
| below college | 9438 (48.58) | 1.32 (1.25 ~ 1.41) | <.001 |  |
| college Graduate or above | 9989 (51.42) | 1.39 (1.28 ~ 1.51) | <.001 |  |
| **Marital status** |  |  |  | 0.072 |
| live together | 12108 (62.33) | 1.42 (1.29 ~ 1.56) | <.001 |  |
| separation | 7319 (37.67) | 1.28 (1.19 ~ 1.37) | <.001 |  |
| **Hypertension** |  |  |  | <.001 |
| No | 11526 (59.39) | 1.58 (1.38 ~ 1.79) | <.001 |  |
| Yes | 7882 (40.61) | 1.29 (1.21 ~ 1.37) | <.001 |  |
| **Diabetes** |  |  |  | 0.015 |
| No | 15570 (81.37) | 1.39 (1.30 ~ 1.49) | <.001 |  |
| Yes | 3564 (18.63) | 1.28 (1.17 ~ 1.39) | <.001 |  |
| HR: Hazard Ratio,  CI: Confidence Interval |  |  |  |  |

In appendix 3.5, among all patients, the HR for edentulism and all-cause mortality was 1.34, with a 95% CI of 1.27 to 1.41, and a P＜0.001, indicating a significant connection between edentulism and mortality due to all-cause, with a markedly increased risk of mortality by all causes in edentulous patients. In age stratification, the P-value for the interaction test was less than 0.001, suggesting a significant interaction of age on the connection between edentulism and all-cause mortality, with notable risk differences across different age groups. In racial stratification, the P-value for the interaction test was 0.001, indicating a significant interaction of race on the relationship between edentulism and all-cause mortality, with significant risk differences among different racial groups. In stratifications for hypertension and diabetes, the interaction test P-values were <0.001 and 0.015, respectively, indicating significant interactions of hypertension and diabetes status on the connection between edentulism and all-cause mortality, with a more pronounced risk in the group without hypertension and diabetes. This suggested that the impact of different population characteristics on the relationship between edentulism and mortality by all causes varies.
